# Supplementary material for: Nonequilibrium pulsed heating freezes sintering of supported metal nanocatalysts
Source: Nat Commun. 2026 Jan 23;17:1828. doi: 10.1038/s41467-026-68539-5 (PMC12920794; doi:10.1038/s41467-026-68539-5)
Supplement: Supplementary file 1 — Supplementary Information [file 41467_2026_68539_MOESM1_ESM.pdf]

Supplementary Information

Nonequilibrium pulsed heating freezes sintering of supported metal nanocatalysts

Jiawei Huang[1], Zhouyang Zhang[2]*, Guangren Wang[1], Jiaqi Chen[1], Yucheng Zhang[1], Jian Zhou[1], Chunxian Xing[1], Yiran Ying[3]*, Changshui Huang[4]*, and Linfeng Fei[1]*

[1]School of Physics and Materials Science, Jiangxi Provincial Key Laboratory of Photodetectors, Jiangxi Engineering Laboratory for Advanced Functional Thin Films, Nanchang University, Nanchang 330031, China

[2]School of Materials and New Energy, Ningxia University, Yinchuan 750021, China

[3]State Key Laboratory of Solidification Processing, Center for Nano Energy Materials, Northwestern Polytechnical University and Shaanxi Joint Laboratory of Graphene (NPU), Xi'an 710072, China

[4]Beijing National Laboratory for Molecular Sciences, Organic Solids Laboratory, Institute of Chemistry, Chinese Academy of Sciences, Beijing 100190, China

*E-mail: zhangzhouyang@nxu.edu.cn; yiranying@nwpu.edu.cn; huangcs@iccas.ac.cn; feilinfeng@gmail.com

Supplementary Figures

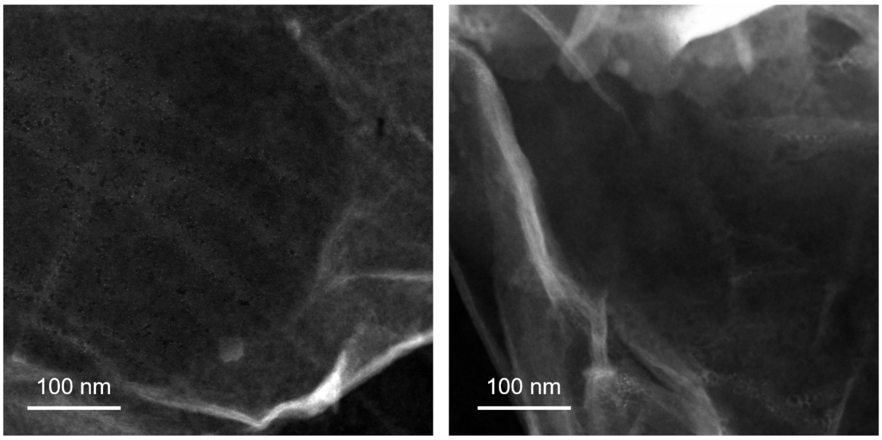

Supplementary Fig. 1 STEM images of graphene flakes after plasma treatment. The images demonstrate that no visible damage occurs during the Ar^{+}/O^{2-} plasma treatment.

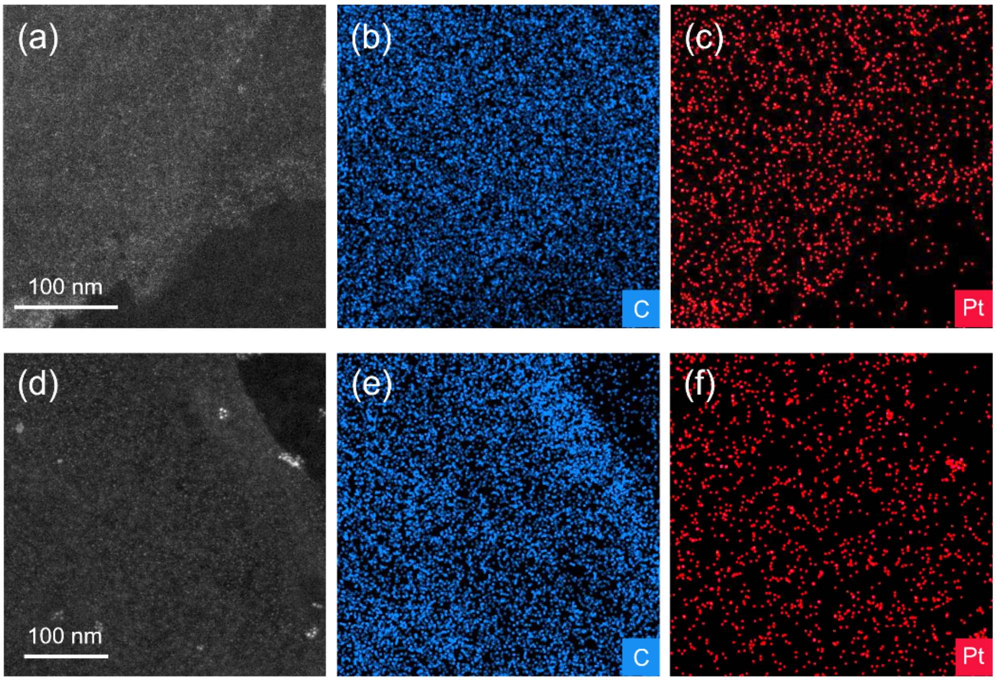

Supplementary Fig. 2 EDS elemental mappings of isolated Pt/graphene flakes for (a-c) conventional heating and (d-f) pulsed heating.

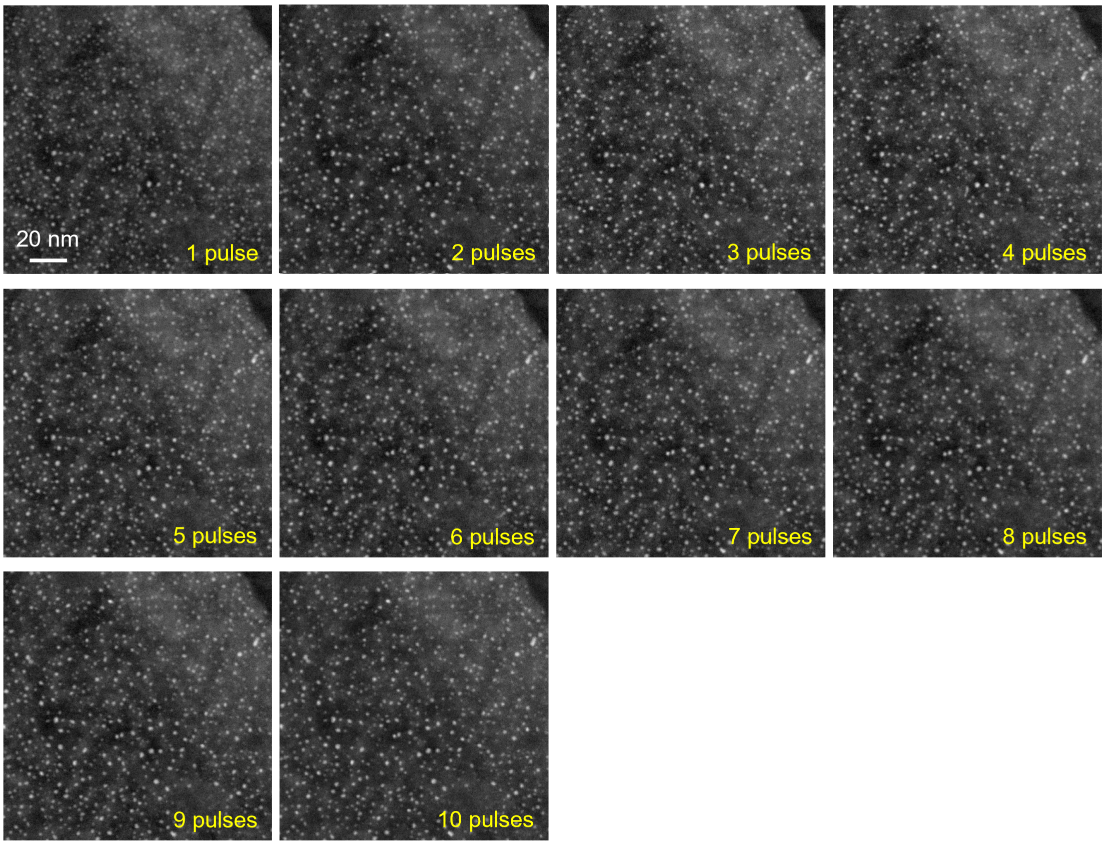

Supplementary Fig. 3 In situ STEM image series showing the evolution of Pt NPs on graphene support with an increasing number of pulses.

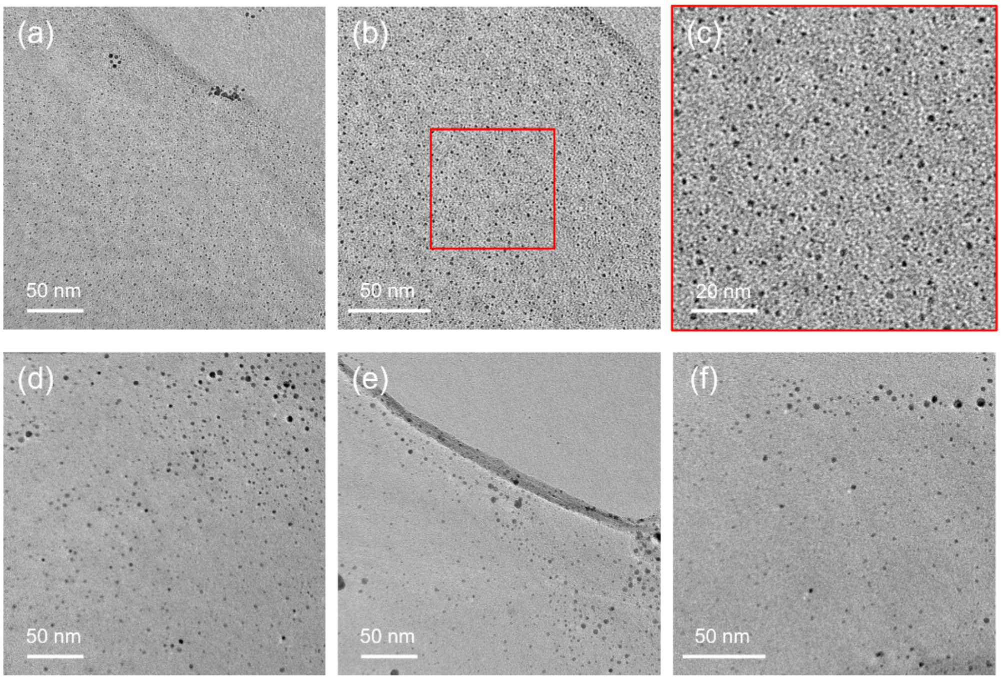

Supplementary Fig. 4 Comparison of particle distributions on graphene support under two heating modes. (a-c) TEM images showing the distribution of Pt NPs on graphene support after 10 heating pulses. (d-f) TEM images showing the distribution of Pt NPs on graphene support after conventional heating to 1000 ºC. The images highlight the significant sintering resistance of Pt NPs under pulsed heating.

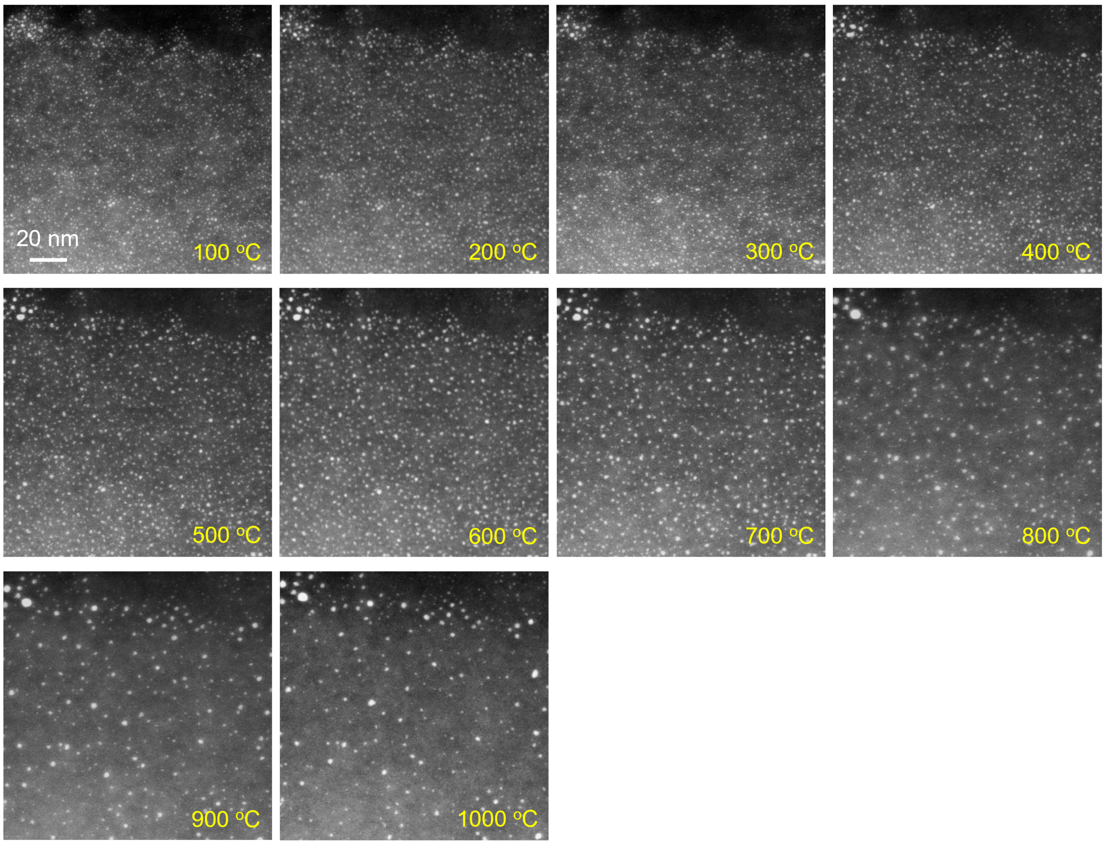

Supplementary Fig. 5 In situ STEM image series showing the evolution of Pt NPs on graphene support against the rising temperature under conventional heating. The images illustrate the progressive agglomeration, sintering and disappearance of Pt NPs as the temperature rises, emphasizing the limitations of conventional heating in maintaining particle stability.

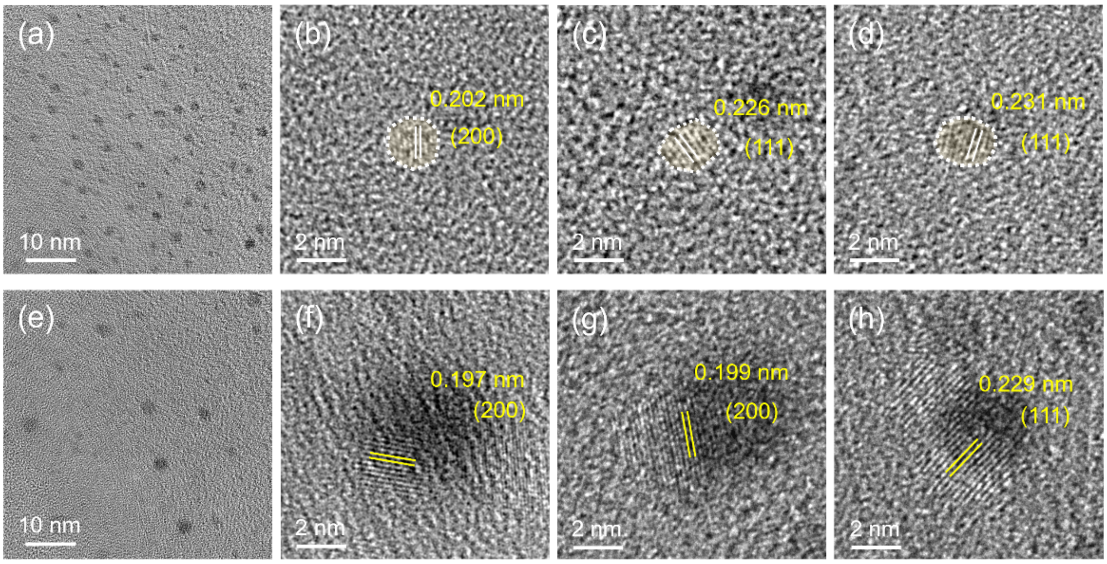

Supplementary Fig. 6 Comparison of crystal structures of Pt NPs under two heating modes. (a-d) Lattice-resolved TEM images showing the crystal structure of Pt NPs after 10 heating pulses. (e-h) Lattice-resolved TEM images showing the crystal structure of Pt NPs after conventional heating to 1000 ^{o}C.

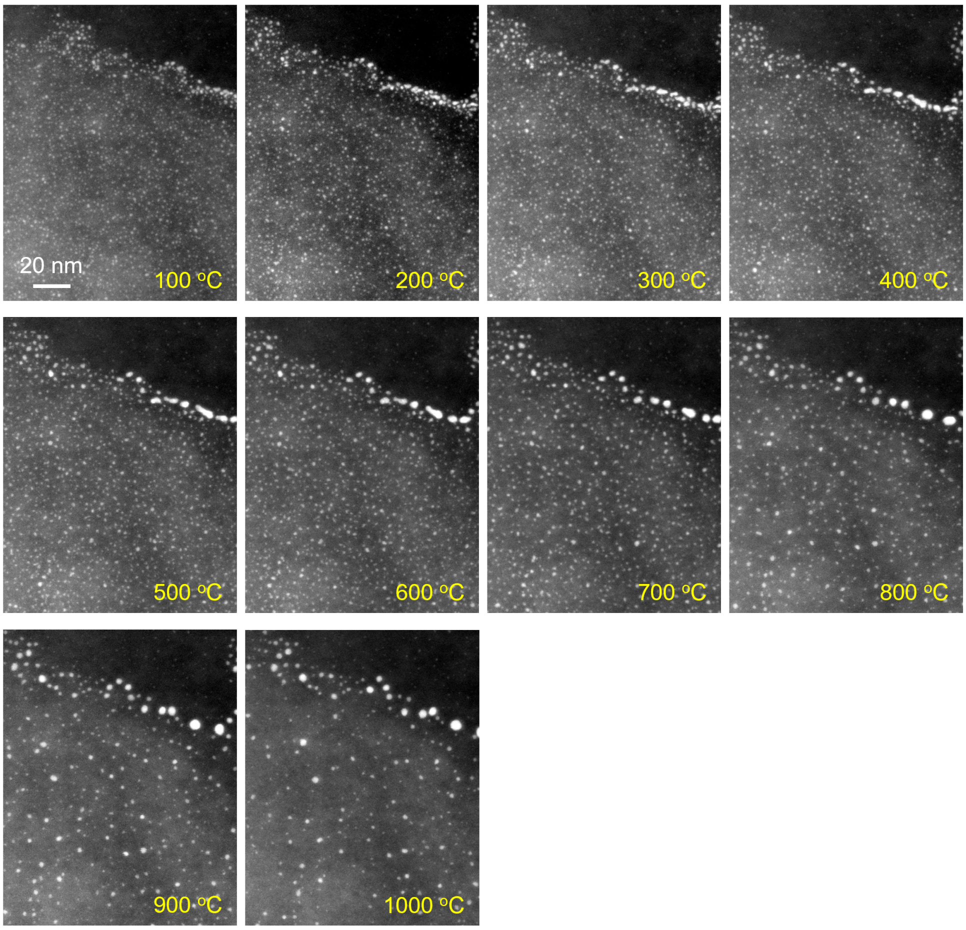

Supplementary Fig. 7 In situ STEM image series presenting an additional set of observations for the evolution of Pt NPs on graphene support under conventional heating mode.

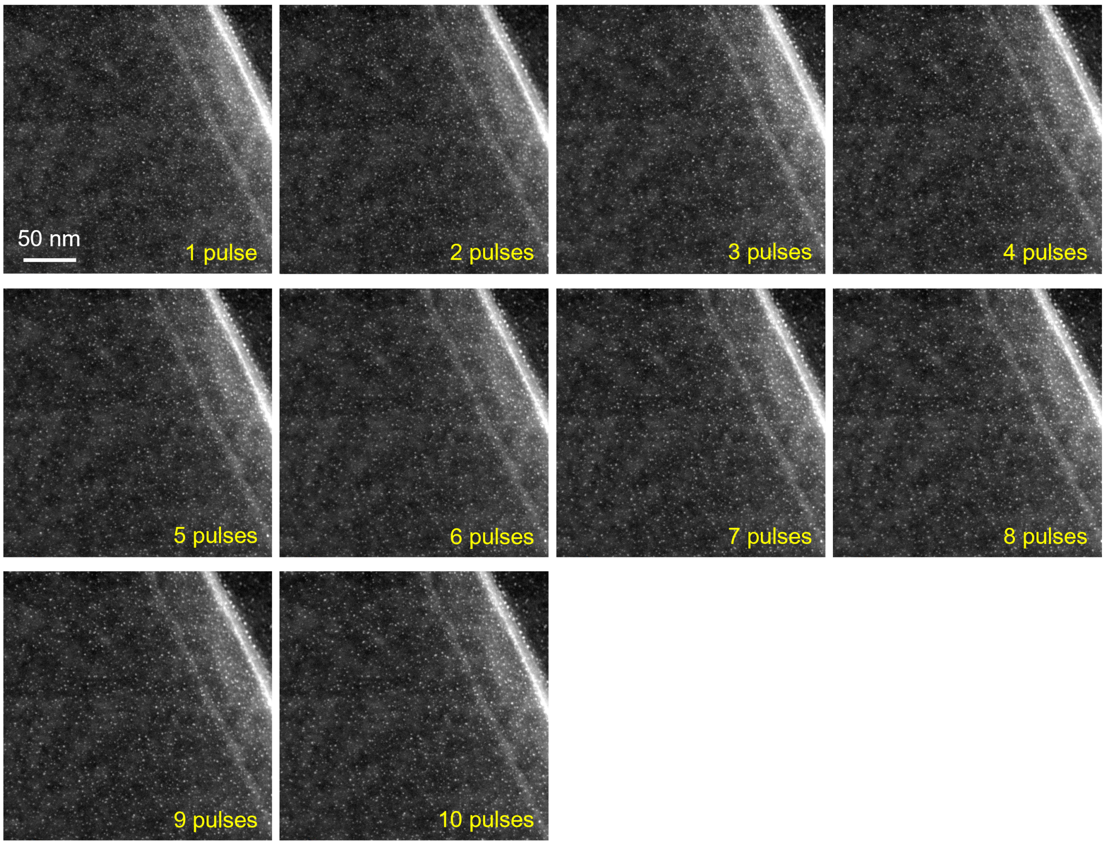

Supplementary Fig. 8 In situ STEM image series presenting an additional set of observations for the evolution of Pt NPs on graphene support during 10 heating pulses.

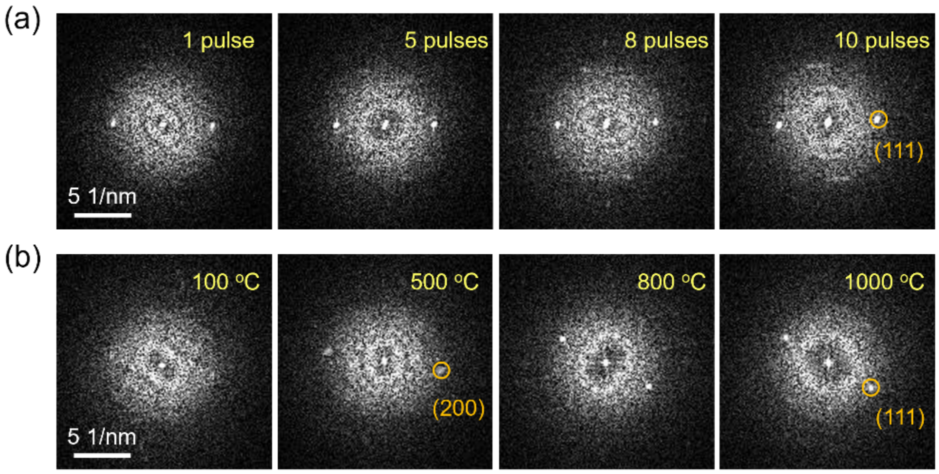

Supplementary Fig. 9 FFT pattern series corresponding to (a) pulsed heating process in Fig. 2k and (b) conventional heating process in Fig. 2l.

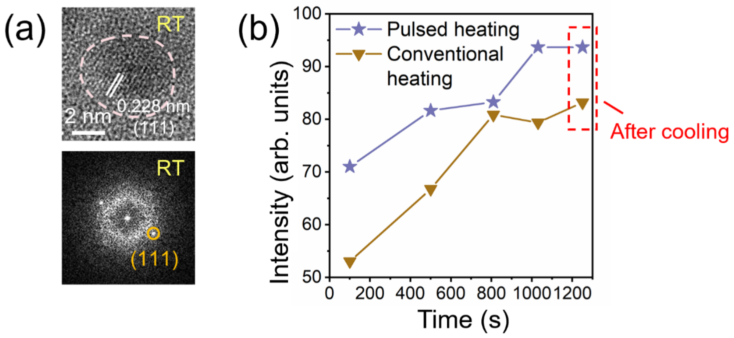

Supplementary Fig. 10 Comparison of relative crystalline orders for Pt NPs under two heating modes. (a) HRTEM image and corresponding FFT pattern of the same Pt NP in conventional heating (**Fig. 2l**) after cooling to RT. (b) Evolutions of relative crystalline orders for Pt NPs throughout the pulsed heating and conventional heating.

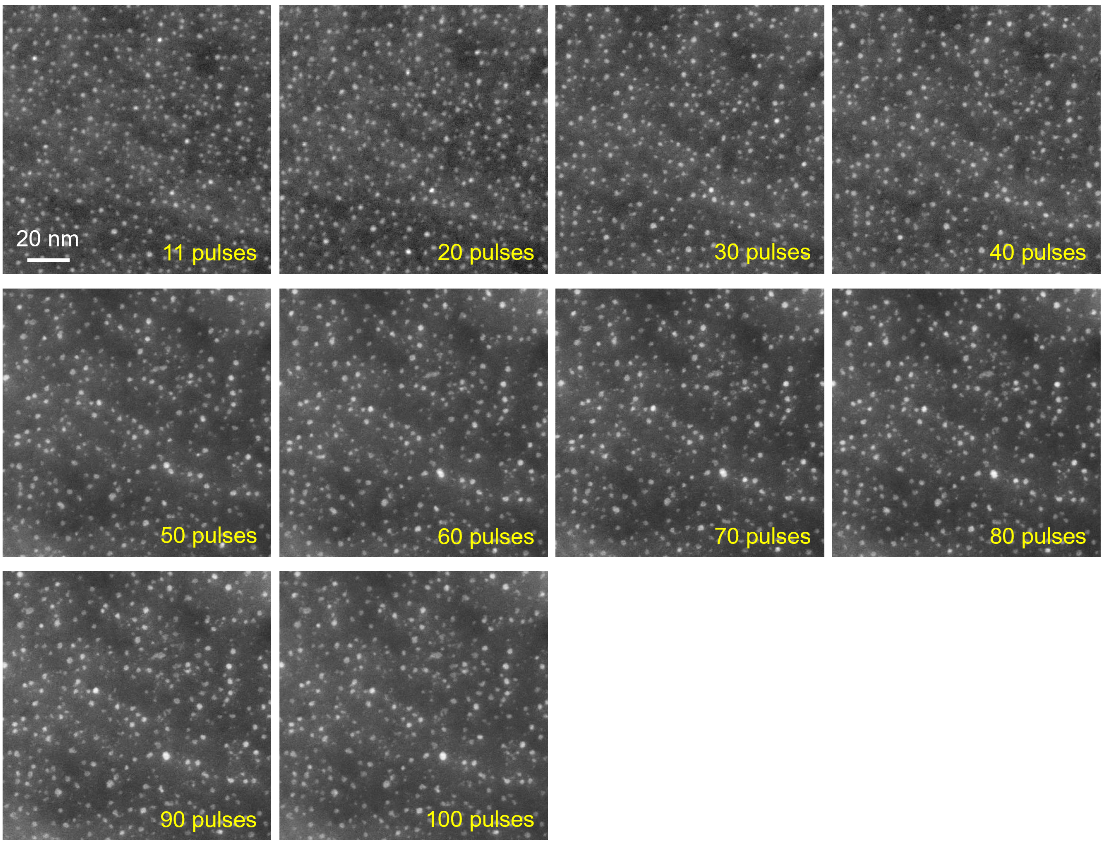

Supplementary Fig. 11 In situ STEM image series illustrating the evolution of Pt NPs on graphene support during 100 heating pulses.

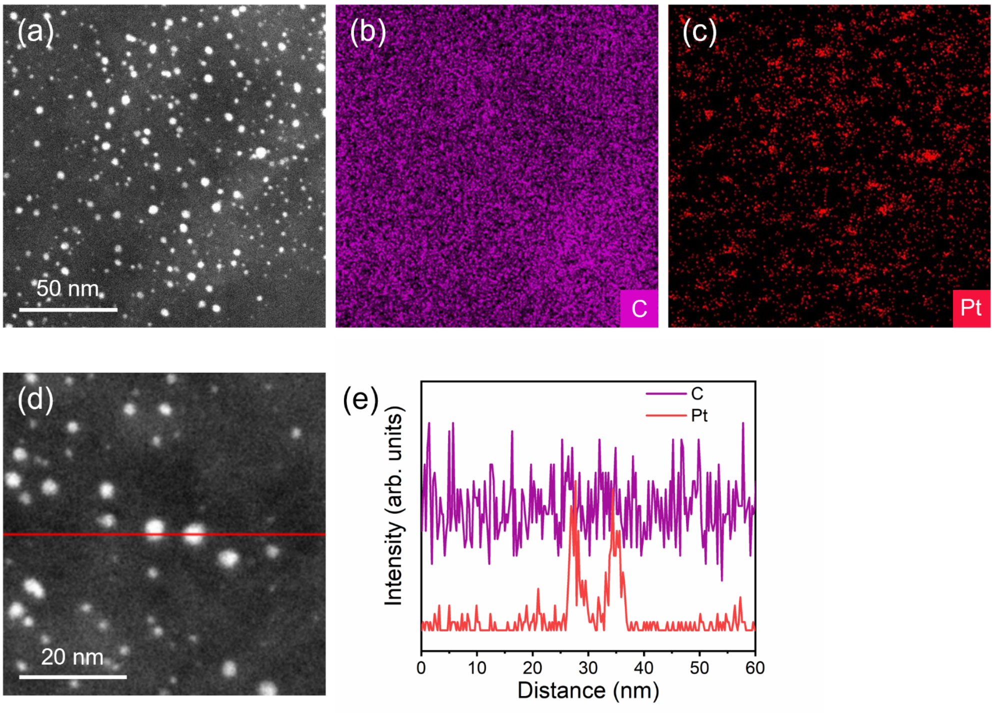

Supplementary Fig. 12 EDS analysis of Pt/graphene interface after 100 heating pulses. (a-c) EDS elemental mappings for Pt/graphene interface after 100 heating pulses, showing the dense and uniform distribution of Pt NPs on graphene support. (d, e) EDS line scan of Pt NPs after 100 heating pulses.

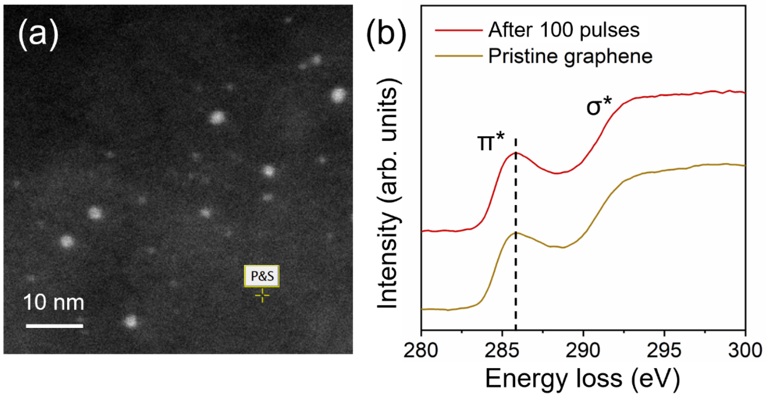

Supplementary Fig. 13 Stability of graphene support under pulsed heating. (a, b) STEM image and C K-edge EELS spectra of graphene support before and after 100 heating pulses.

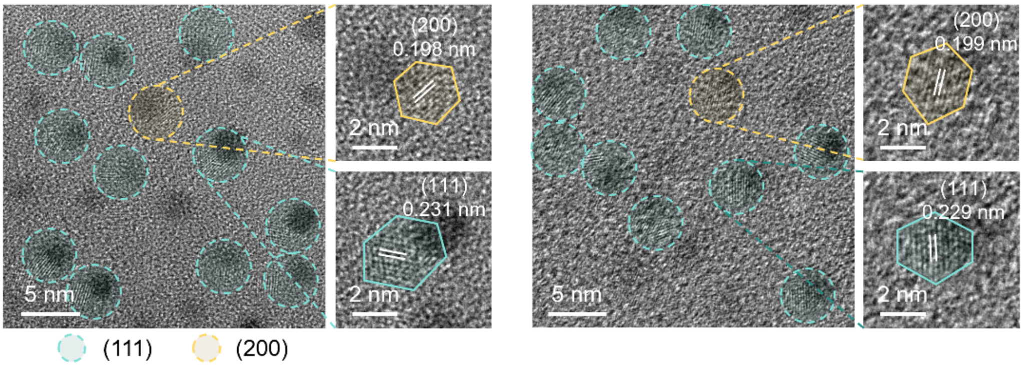

Supplementary Fig. 14 HRTEM images showing the structural analysis of Pt NPs after 100 heating pulses. NPs that exposed their (111) and (200) crystal planes are marked with green and yellow circles, respectively. The result indicates the dominance of the stable (111) crystal plane.

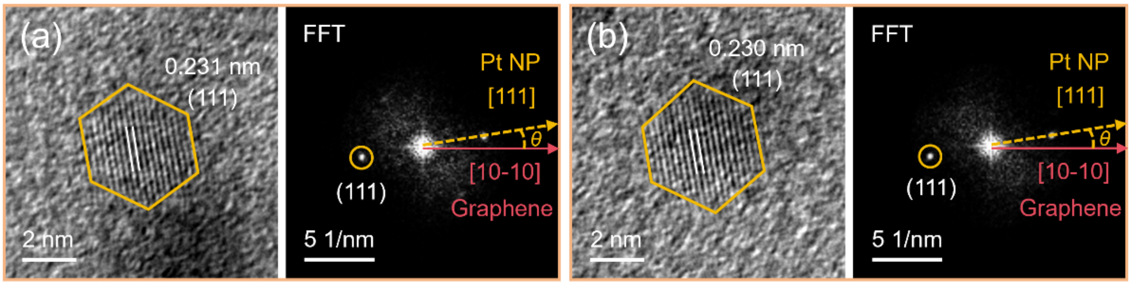

Supplementary Fig. 15 Determination of orientation angle between Pt NPs and graphene support. (a, b) HRTEM images and corresponding FFT pattern of Pt NPs supported on graphene support, showing their crystallographic orientation relationships against graphene flakes.

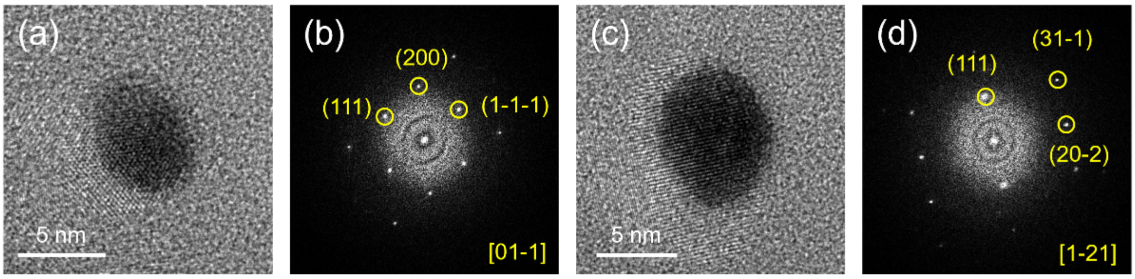

Supplementary Fig. 16 Structural analysis of Pt NPs after conventional heating. HRTEM images and corresponding FFT patterns of Pt NPs along the (a, b) [01-1] and (c, d) [1-21] zone axis after conventional heating.

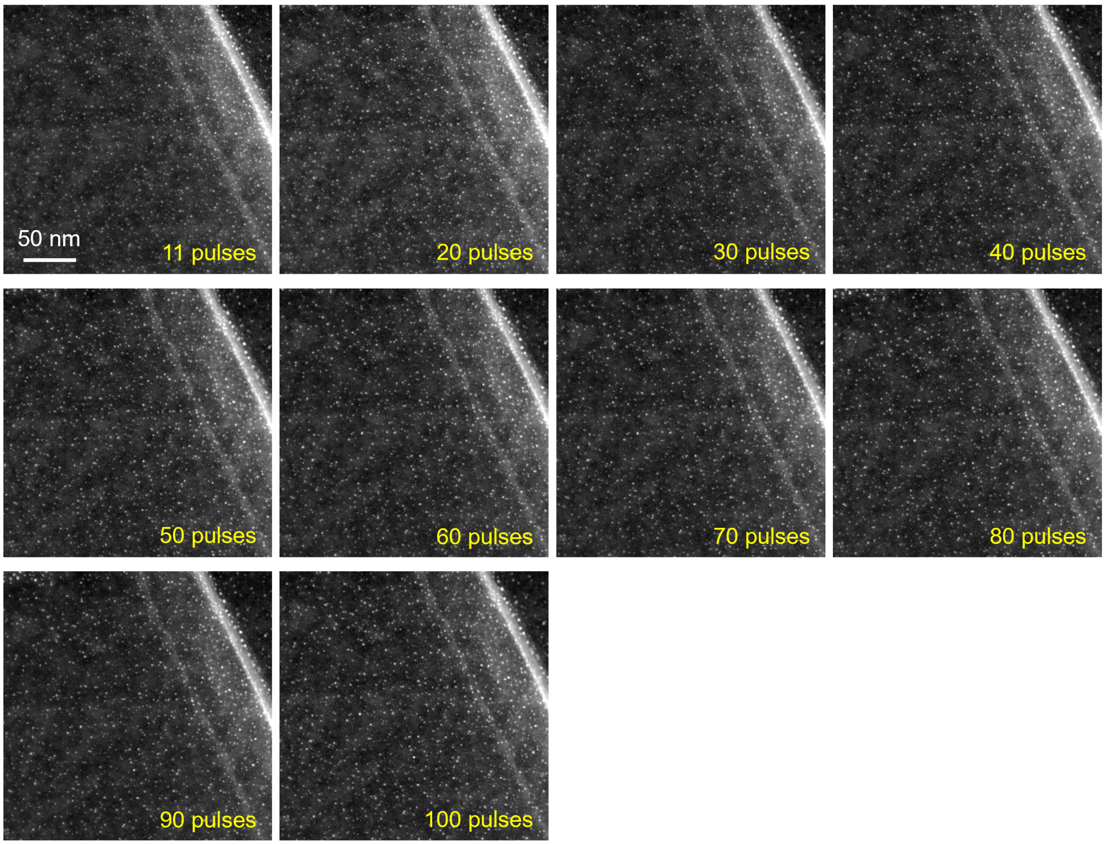

Supplementary Fig. 17 In situ STEM image series showing another set of evolution for Pt NPs on graphene support during 100 heating pulses.

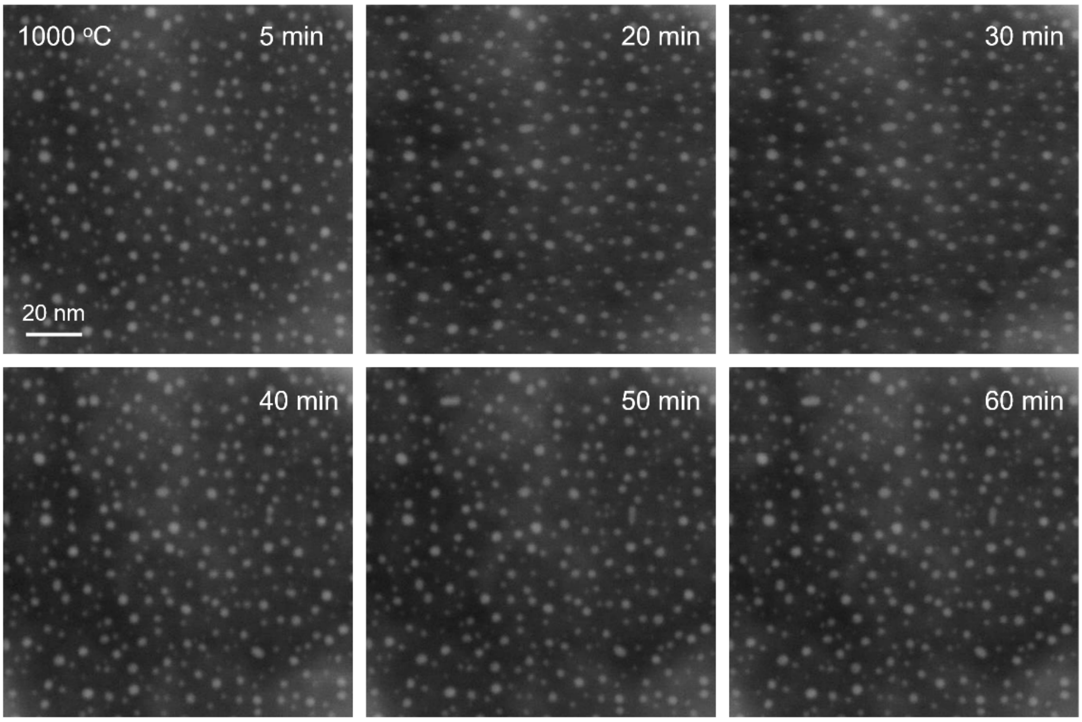

Supplementary Fig. 18 In situ STEM images showing the time-lapsed thermal stability of Pt/graphene nanocatalyst formed after 100 heating pulses at 1000 °C.

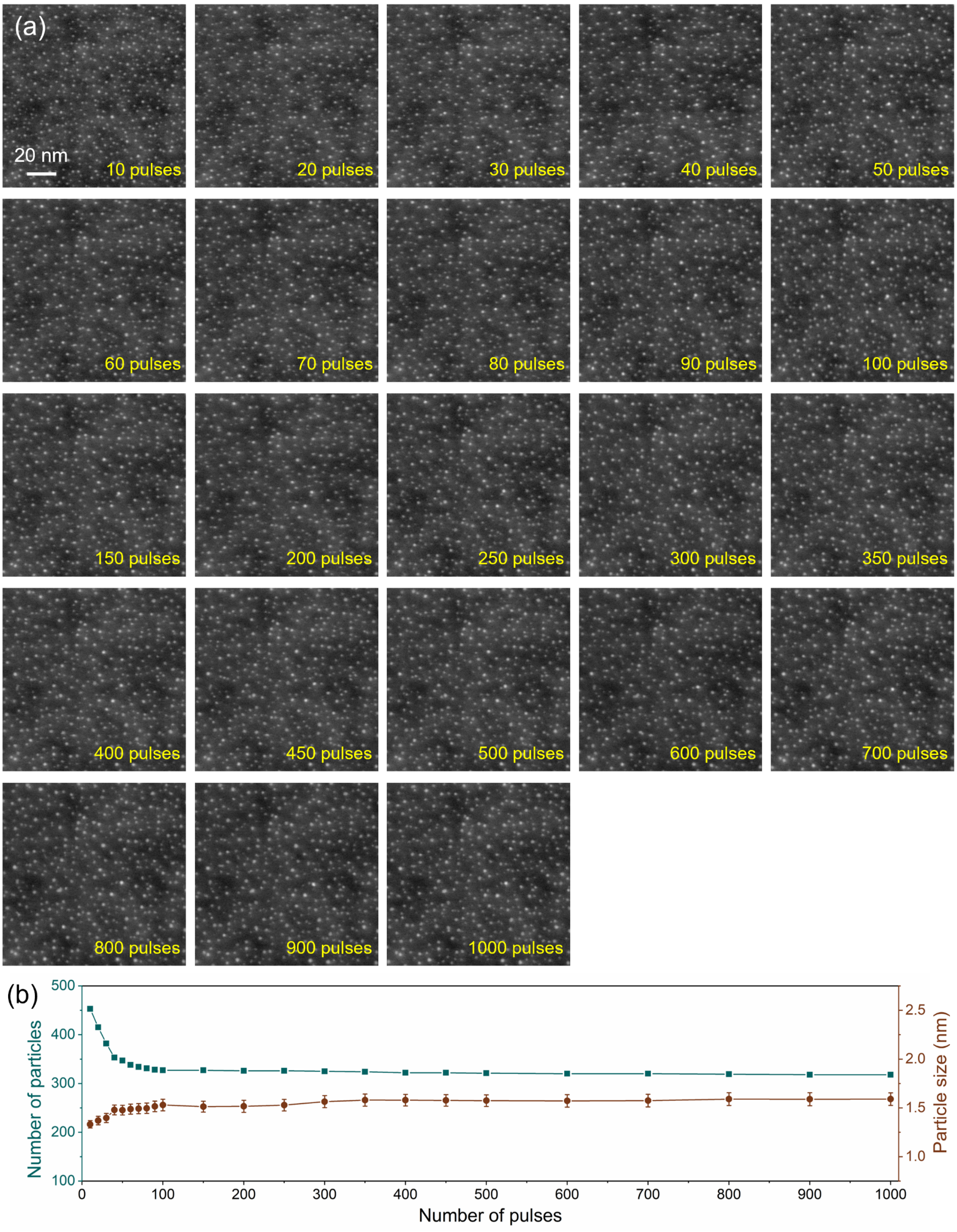

Supplementary Fig. 19 Microscopic sintering behaviors of Pt NPs under 1000 heating pulses. (a) In situ STEM image series showing the evolution for Pt NPs on graphene support during 1000 heating pulses, with a heating/cooling rate of 150 °C s^{-1} and a peak temperature duration at 1000 °C for 1 s. (b) Number and average size of Pt NPs as functions of pulse number for (a). Error bars represent the 95% confidence interval of the mean.

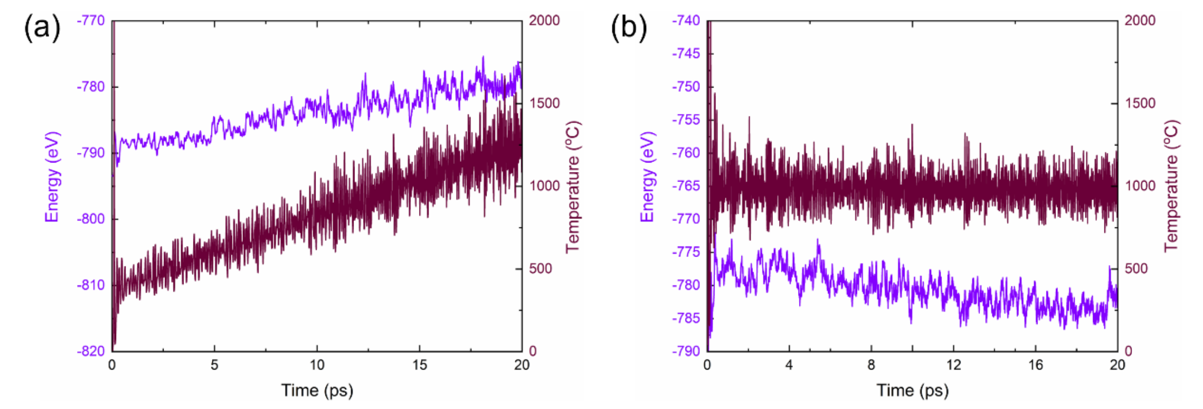

Supplementary Fig. 20 Temperature and energy as functions of simulation time during AIMD simulations for Pt/graphene structure under (a) pulsed heating and (b) conventional heating.

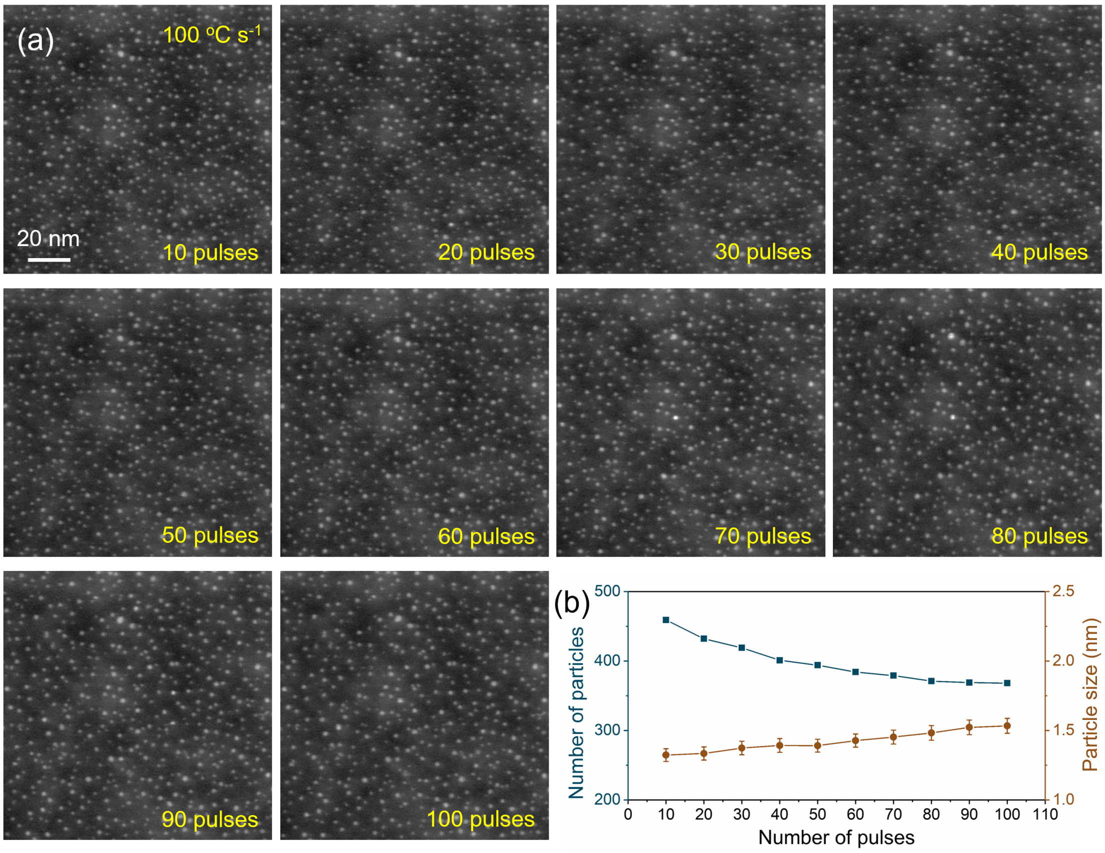

Supplementary Fig. 21 Microscopic sintering behaviors of Pt NPs under pulsed heating with a heating/cooling rate of 100 °C s^{-1}. (a) In situ STEM image series showing the evolution for Pt NPs on graphene support during 100 heating pulses, with a heating/cooling rate of 100 °C s^{-1}. (b) Number and average size of Pt NPs as functions of the pulse number for (a). Error bars represent the 95% confidence interval of the mean.

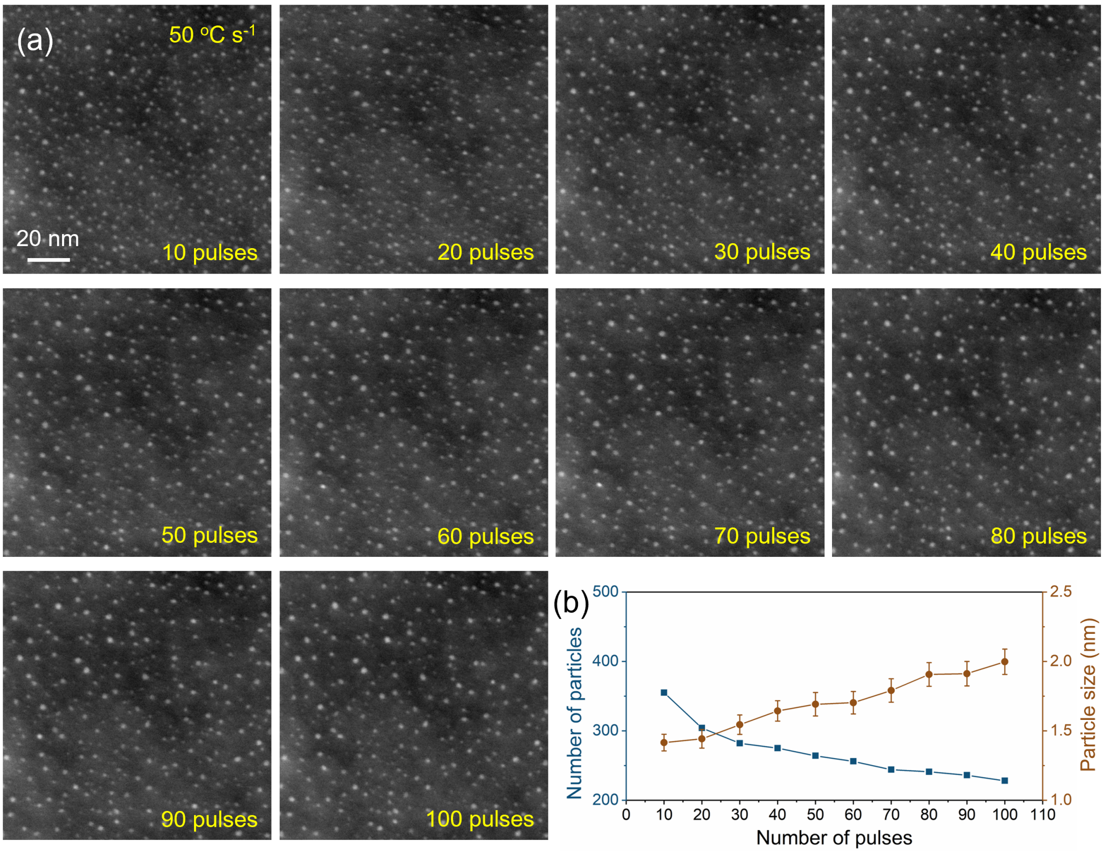

Supplementary Fig. 22 Microscopic sintering behaviors of Pt NPs under pulsed heating with a heating/cooling rate of 50 °C s^{-1}. (a) In situ STEM image series showing the evolution for Pt NPs on graphene support during 100 heating pulses, with a heating/cooling rate of 50 °C s^{-1}. (b) Number and average size of Pt NPs as functions of the pulse number for (a). Error bars represent the 95% confidence interval of the mean.

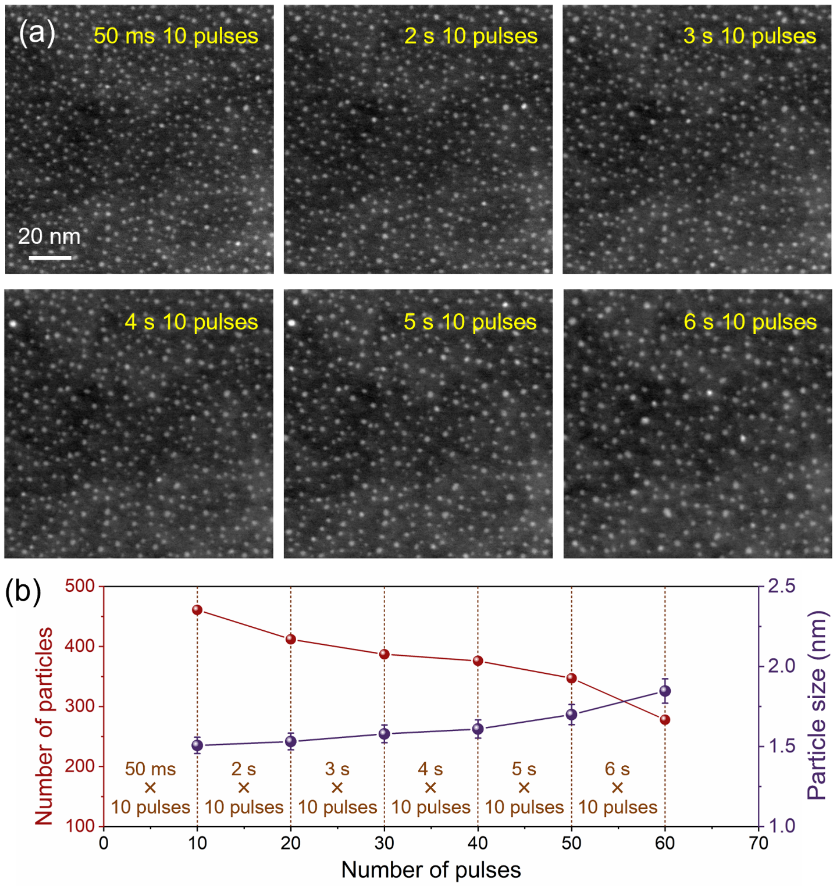

Supplementary Fig. 23 Microscopic sintering behaviors of Pt NPs under pulsed heating with varying durations. (a) In situ STEM image series showing the evolution for Pt NPs on graphene support during 60 heating pulses, with a varying duration of 50 ms, 2 s, 3 s, 4 s, 5 s, and 6 s for each duration within 10 pulses (heating/cooling rate: 150 $^{o}C\ s^{-1}$). (b) Number and average size of Pt NPs as functions of the pulse number for (a). Error bars represent the 95% confidence interval of the mean.

Supplementary Discussion

Using the Pt/graphene system as an example, we define the kinetic boundaries in which pulsed heating suppresses NP sintering. In situ STEM experiments show that heating/cooling rates ≥100 °C s^{-1} stabilize Pt NPs over 100 pulses, whereas slower rates allow coarsening (**Supplementary Figs. 21** and **22**). Pulse durations ≤4 s at 1000 °C preserve progressive convergence of particle size and number, while longer pulses trigger rapid coalescence (**Supplementary Fig. 23**). Besides, the temperature window lies between the precursor decomposition (~400 °C) and the size-dependent Pt melting point (~1005 °C), with sintering effectively suppressed even near 1000 °C if rate and duration criteria are met (**Supplementary Fig. 21**). Pulse number does not affect stability, although additional pulses further improve crystallinity and metal-support bonding (**Supplementary Fig. 19**). These results establish a clear kinetic mechanism and provide design rules for NP stabilization via pulsed or temporally modulated heating: rates ≥100 °C s^{-1}, dwell ≤4 s, and temperatures between decomposition and melting.
